# Is Technology Enhancing or Hindering Interpersonal Communication? A Framework and Preliminary Results to Examine the Relationship Between Technology Use and Nonverbal Decoding Skill

**DOI:** 10.3389/fpsyg.2020.611670

**Published:** 2021-01-15

**Authors:** Mollie A. Ruben, Morgan D. Stosic, Jessica Correale, Danielle Blanch-Hartigan

**Affiliations:** ^1^Department of Psychology, University of Maine, Orono, ME, United States; ^2^Center for Healthcare Organization and Implementation Research, VA Boston Healthcare System, Boston, MA, United States; ^3^Department of Natural and Applied Sciences, Bentley University, Waltham, MA, United States

**Keywords:** technology, nonverbal communication, decoding ability, interpersonal accuracy, communication skills

## Abstract

Digital technology has facilitated additional means for human communication, allowing social connections across communities, cultures, and continents. However, little is known about the effect these communication technologies have on the ability to accurately recognize and utilize nonverbal behavior cues. We present two competing theories, which suggest (1) the potential for technology use to *enhance* nonverbal decoding skill or, (2) the potential for technology use to *hinder* nonverbal decoding skill. We present preliminary results from two studies to test these hypotheses. Study 1 (*N* = 410) found that global screen time was unrelated to nonverbal decoding skill. However, how participants spent their time using technology mattered. Participants who reported more active technology use (i.e., posting content) self-reported that their nonverbal decoding skill (as measured by the Emotional Sensitivity subscale of the Social Skills Inventory) was superior but performed worse on objective measures of decoding skill (using standardized tests including the Diagnostic Analysis of Nonverbal Accuracy-Adult Faces and the Workplace Interpersonal Perception Skill). By contrast, passive users performed significantly better on objective measures of nonverbal decoding skill; although they did not self-report any difference in their skill compared to less passive users. Study 2 (*N* = 190), and a mini-meta analysis of both studies, replicated this pattern. These effects suggest a roadmap for understanding the theoretical relationship between technology use and nonverbal communication skills. We also provide recommendations for future research, including the use of experimental designs to determine causal pathways and to advance our conceptual understanding of the relationship between technology use and nonverbal decoding skill.

## Introduction

A young-professional is woken up to the sound of a buzzing alarm, and grudgingly rolls over to grab their phone. Perhaps this individual begins their morning by passively scrolling through their Facebook feed in order to determine their colleague’s reaction to the heated presidential debate the night before. Or maybe they snap a quick picture of their #OOTD (i.e., Outfit of the Day) to send to their close friend. After returning home from a long day of work-based videoconference calls, this individual may spend the next few hours sucked into the whereabouts of their favorite social media influencer, or casually swiping through some dating profiles. Before retiring to bed, however, they make sure to post a quick inspiring quote to their Twitter profile.

This scenario, while fictitious, illustrates the increasing relationship many individuals have with technology from the instant they wake up, to the instant they go to bed. Technology serves various functions, from increasing office productivity, facilitating big data collection, enhancing record keeping, and above all else, providing a distinctly digital way for humans to *communicate* with one another. Indeed, the rate of communicative instances via technology per day in 2020 is astounding: 350 million photos uploaded to Facebook, 500 million tweets, 3 billion snapchats, and over 26 billion texts by Americans alone (Aslam, 2020a,b; Sayce, 2020; Tocci, 2020).

While the digital revolution has certainly changed the way individuals can communicate, little empirical results exists regarding the effect of technology on an individual’s communication skills. Specifically, because technology markedly changes the available information individual’s use to decode the communicative intents of others (e.g., determining a friend’s emotional state via short text message instead of their facial expression), are those who spend large quantities of time communicating online better or worse decoders of nonverbal information? Not only is nonverbal decoding a crucial component of general social and communication skills, but it has been tied to better interpersonal outcomes (e.g., Hall et al., 2009), can be easily assessed with validated, reliable, and standardized objective measures, and can be improved with practice and feedback trainings (e.g., Schlegel et al., 2017b). Therefore, the question of whether technology may affect nonverbal decoding, or how accurately a perceiver can recognize and interpret the nonverbal behaviors of another person, is important to empirically address.

Supplementing or even fully replacing face-to-face communication with technology-mediated communication affects both the *number* of nonverbal cues, as well as the *types* of nonverbal cues that individuals use to decode communicative meaning (Vinciarelli, 2017). For example, text messages may not allow access to important vocal cues (e.g., pitch, tone, inflections), but may have distinct timing and spacing cues to draw from Döring and Pöschl (2008). By contrast, video conferencing technologies may allow access to vocal cues, but may limit the ability to engage in mutual eye gaze or perceive body movements and gestures (Ferrán-Urdaneta and Storck, 1997; Neureiter et al., 2013). If individuals rely more heavily on technology-mediated, as opposed to face-to-face, interactions as a primary means of communication, it seems likely that the nonverbal decoding skill individuals ordinarily employ in face-to-face communication would be impacted (e.g., worsened, or perhaps enhanced).

This paper applies communication skills theories and conceptual accounts of technology use to examine the role of technology use on an individual’s ability to accurately perceive the nonverbal behavior displayed by others (i.e., nonverbal decoding skill). For the purposes of this paper, we define technology use as any technology or application on a smart phone that contributes to *communication* online (e.g., use of social media sites, texting, emailing). Cell phone use is the predominant method of technology use by young adults in the United States today with 96% of 18–26 years-old young adults reporting ownership of a smart phone (Pew Research Center., 2019). Therefore, for the remainder of the paper, when discussing technology use, we are referring specifically to smart phone use.

We start by reviewing two competing hypotheses, that technology use either enhances or hinders communication skills. We then present results from two cross-sectional studies and a mini meta-analysis of these studies on the relationship between technology use and nonverbal decoding skill to inform our understanding of which of the competing hypotheses is more likely supported. Finally, we make recommendations for future research aimed at disentangling the causal relationship between technology use and nonverbal decoding skill.

## Technology Use May Enhance Communication Skills

The most effective way to improve nonverbal decoding skill is by practicing decoding nonverbal cues and receiving feedback on the accuracy of one’s perceptions (Blanch-Hartigan et al., 2012; Schlegel et al., 2017a). Regarding the relationship between technology use and nonverbal decoding skill, some theorists have argued that technology-mediated communication may enhance communication skills by providing a safe environment to practice sending and receiving nonverbal cues, and allowing for feedback regarding the accuracy of one’s perceptions (e.g., Stritzke et al., 2004; Ellison et al., 2007; Valkenburg and Peter, 2009). Because it is unusual in face-to-face interactions to receive feedback about one’s decoding ability, it may be that spending more time using technology to interact with others may facilitate face-to-face interactions by providing this type of practice and feedback to users on a regular basis.

### Liberated Relationship Perspective

One hypothesis which falls into this “enhancement” framework is the Liberated Relationships Perspective (Hu et al., 2004). This theory argues that increased internet usage has allowed individuals who may not typically engage in conversation the opportunity to engage with one another through technology-mediated communication. Some of the constraints may be psychological, such as in cases of shyness and social anxiety (Stritzke et al., 2004), or physical, such as in cases of distant geographical locations (Ellison et al., 2007). According to this framework, internet usage may afford an increase in the *number* of interactions an individual is able to engage in. If the internet supplements, instead of detracts from, face-to-face interactions, individuals may have increased opportunities to practice nonverbal decoding with a greater number and variety of communication partners.

### Internet Enhanced Self-Disclosure Hypothesis

While not directly related to communication skill, the Internet Enhanced Self-Disclosure Hypothesis also provides support for improved nonverbal decoding skill with increased technology use (Valkenburg and Peter, 2009). This theory posits that greater technology use may enhance social connectedness and wellbeing by enhancing *online self-disclosure*. The authors define online self-disclosure as “online communication about personal topics that are typically not easily disclosed, such as one’s feelings, worries, and vulnerabilities” (p. 2). Because online platforms allow for the sharing of intimate information to a significantly greater degree than do face-to-face interactions, it is likely that individuals are afforded more opportunities to practice decoding and receive feedback regarding affective information. Individuals who engage in technology-mediated communication more frequently may become more skilled decoders of nonverbal information, perhaps for affective information in particular.

## Technology Use May Hinder Communication Skills

While these two “enhancement” theories describe the ways in which increased technology usage may allow individuals more opportunities to practice decoding nonverbal communication, others have argued a competing perspective. Specifically, researchers have argued that technology may hinder specific communication skills. Spending time communicating via technology may result in less face-to-face interactions and therefore less practice decoding nonverbal information in whole, as well as from specific cue channels (e.g., vocal tone) which are reduced or absent in many technology platforms (Kraut et al., 1998; Nie, 2001; Patterson, 2019). In this way, the type of communication skills learned or practiced in technology-mediated communication are not equivalent to, and may even hinder, the skills required to decode nonverbal behavior in face-to-face interactions.

### Reduction Hypothesis

In the early 1990s, several researchers theorized that the internet had detrimental effects on adolescent wellbeing and social connectedness (Kraut et al., 1998; Nie, 2001). It was assumed that because the internet motivates adolescents to form superficial online relationships with strangers that are less beneficial than their real-world relationships, time spent online occurs at the expense of time spent with existing relationships. The Reduction Hypothesis posits that it is the lack of or decrease in face-to-face interacting that leads to detrimental communicative consequences rather than technology itself (Valkenburg and Peter, 2009).

Valkenburg and Peter (2009) propose two important updates to this theory based on changes in how individuals use the internet to communicate since the Reduction Hypothesis was first introduced. First, in the second half of the 1990s, it was hard to maintain a pre-existing social network on the internet because not a lot of people had access to it, often resulting in online friends separate from offline friends. Today, with more widespread access and utilization of the internet and social media, individuals spend more time online connecting with people they also spend time with in face-to-face interactions as opposed to forming online-only relationships with strangers (Valkenburg and Peter, 2009). However, the communication skills, such as nonverbal decoding, that individuals develop through online interactions may not translate to actual face-to-face interactions. As such, time spent online may stunt the development of nonverbal decoding necessary for face-to-face interactions. Therefore, although our internet habits have changed, the Reduction Hypothesis is still relevant to theorizing regarding the effects of technology use on nonverbal decoding ability.

### Cues-Filtered–Out Theory

In addition to reducing the amount of time individuals spend interacting face-to-face, theorists have also noted that many technology-mediated communication platforms greatly reduce both the number as well as the kinds of nonverbal cues technology users are exposed to. Cues absent from some technology-mediated communication (e.g., social media, texting, emailing) can include physical appearance, tone of voice, facial expression, gaze, posture, touch, space, and gestures (Kiesler et al., 1984; Siegel et al., 1986). These nonverbal cues are important in expressing relative status, affect, relationship roles, and many other interpersonal dimensions. This Cues-Filtered-Out Theory (Culnan and Markus, 1987; Sproull and Kiesler, 1986) suggests that without these cues available, especially for low bandwidth technology (i.e., communication systems with access to only one or two channels such as vocal, kinesics, or proxemics), certain communicative functions are lost. Although higher bandwidth systems may allow for certain nonverbal cues, these cues are often more obvious and lack complexity, which may cause individuals to lose the ability to decode more subtle nonverbal cues (e.g., facial expressions are more complex than emoji’s, vocal intensity is more complex than CAPITALIZING words). Therefore, this theory suggests that the filtering out of important nonverbal cues (e.g., especially for individuals who use low bandwidth technology systems) impacts an individual’s ability to receive practice and feedback on the accuracy of their nonverbal decoding attempts, thereby hindering nonverbal decoding skill (Walther and Parks, 2002).

## Current Research and Hypotheses

The primary objective of the current research is to empirically examine the relationship between technology use and nonverbal decoding skill via two studies and a mini meta-analysis combining results from these two studies. Because individuals may use technology the same amount but differ in *how* they spend their time online, we measured users’ online communication activity via objective global screen time use taken from iPhone users, as well as the degree of self-reported active technology use (posting selfies and photographs, responding to others’ posts) and the degree of self-reported passive technology use (scrolling through photographs and others’ posts but not responding or posting themselves). In addition, we also sought to be thorough in our assessment of nonverbal decoding skill, as researchers have demonstrated that there are different *kinds* of decoding skills subsumed by a higher-order global decoding skill (Schlegel et al., 2017a). Therefore, we employed three distinct measures of nonverbal decoding, two objective assessments of skill using a standardized, validated, and reliable test of emotion recognition [i.e., Diagnostic Analysis of Nonverbal Accuracy-Adult Faces (DANVA-2AF; Nowicki and Duke, 1994)] and a newly developed test that assesses relevant decoding ability in the workplace such as inferring behavioral intentions, personality traits, status, interpersonal attitudes (dominance/cooperativeness and motivations), behavioral outcomes, and thoughts and feelings [i.e., the Workplace Interpersonal Perception Skill (WIPS; Dael et al., in preparation)], and one self-report measure [the Emotional Sensitivity subscale of the Social Skills Inventory (SSI; Riggio, 2005)]. Together, we utilized these various measures of technology and nonverbal decoding skill in order to test the preceding competing hypotheses: (1) more technology use is related to better nonverbal decoding skill vs. (2) more technology use is related to poorer nonverbal decoding skill.

## Materials and Methods

### Study 1

#### Participants

Data were collected from 410 participants in the University of Maine introductory participant pool for a study on perceiving nonverbal signals in others. Of these, 51% were male and 48% were female. A total of 377 (92%) participants identified as white, 15 (4%) as Asian, 14 (3%) as American Indian or Alaska Native, 12 (3%) as Black, 2 (0.5%) as Native Hawaiian or Pacific Islander, and 33 (8%) as Other. Their ages ranged from 18 to 29 (*M* = 19.09, *SD* = 1.56). A power analysis conducted using G^∗^Power (Faul et al., 2007) assuming a small to medium effect (*r* = 0.15) of technology use on nonverbal decoding skill indicated that 343 participants would be needed to achieve 80% power using an alpha level of 0.05 (two-tailed). The final sample of participants exceeds this threshold, indicating that the present study is sufficiently powered to detect small to medium effects.

#### Measures

##### Technology Use

Three separate measures of technology use were collected from participants. For iPhone users, participants were instructed to navigate to their phone settings and extract their average daily screen time over the last 7 days in minutes (*N* = 263). This screen time metric is a real-time report of how much time a participant spends with their phone screen *turned on* in an average week (i.e., listening to music with one’s screen off is not included). To ensure participants did not alter their responses in order to appear more socially desirable, we also required that they upload a screenshot of this information. In addition to this objective measure of technology use, participants were asked to self-report on a scale of 0–10 from “does not describe me at all” to “describes me very well” how well the following statements described their technology use, “I tend to be an active user, posting frequently” and “I tend to be a passive user, scrolling through posts and photos.” These two questions comprised our self-report measures of technology use: the degree to which a participant endorsed themselves as an active user separately from the degree to which a participant endorsed themselves as a passive user. Because active user endorsement and passive user endorsement were single item questions rather than a single bipolar item, participants could report any combination of active and passive technology use. That is, a participant could endorse a high degree of active use and a high degree of passive use, they could report a low degree of both, or a high degree of one and not the other. For all analyses, we entered both continuous variables to examine how the independent contribution of active and passive use predicted our outcomes of interest.

##### Nonverbal Decoding Measures

The newly developed WIPS test (Workplace Interpersonal Perception Skill; Dael et al., in preparation; a = 0.67) assesses multiple aspects of decoding skill using 41 brief video segments with and without sound from three types of role-played workplace interactions: a recruiter-applicant negotiation, a helpdesk trouble-shooting scenario, and a company team meeting. Each segment is paired with a multiple-choice question for which the correct answer was based on actual behavior (what happened in the interaction during or after the video segment), instructions that the actors received (e.g., to be competitive), actors’ self-reported personality, or post-interaction evaluations (e.g. perceptions of the other as competitive) and response options varied from 2 options to 6 options depending on the item. In this way, participants must decode multiple simultaneous nonverbal cues (e.g., tone of voice, facial expression) in order to accurately assess the interpersonal characteristics of any given situation. For some items, the video consisted of multiple short segments (e.g., You will see the same person in two different negotiations signing a contract. In which negotiation did the person negotiate the better deal for herself?) while other videos were based off of just one video (e.g., In the following video, you will see 6 people enter the room for a team meeting. Who is the team leader?). Accuracy is calculated as the proportion correct responses compared against a criterion or correct response for each segment.

Participants also completed the Diagnostic Analysis of Nonverbal Accuracy-Adult Faces (DANVA-2AF; Nowicki and Duke, 1994; a = 0.60), a test of emotion recognition ability using static and posed photographs. This measure presents 24 photographs of adult faces with high and low intensity portrayals of the four basic emotions of happiness, anger, sadness, and fear. Accuracy was calculated as the proportion correct.

Finally, participants completed the Emotional Sensitivity (ES; a = 0.80) subscale of the Social Skills Inventory (SSI; Riggio, 2005). The ES subscale consists of 15 self-report items, with a 5-point response scale ranging from “Not at all like me” to “Exactly like me.” The ES subscale specifically assesses self-reported skill for decoding emotional and other nonverbal messages (e.g., *I always seem to know what people’s true feelings are no matter how hard they try to conceal them)*. For analysis purposes, a sum was calculated across items.

### Study 2

Our second study was an exact replication of Study 1 launched approximately 3 months after Study 1 with data from 190 participants from the University of Maine introductory participant pool. Because we had not hypothesized *a priori* the effect of active and passive technology use on nonverbal decoding skill, we wished to collect a second sample of participants in order to investigate whether the pattern of results we describe in Study 1 would replicate. The demographics of this second sample were comparable to those from our first study, with 91 male participants (48%) and 99 females (52%). Of these, 179 (94%) identified as white, 9 (5%) as Asian, 5 (3%) as Black, 2 (1%) as American Indian or Alaska Native, 1 (0.5%) as Native Hawaiian or Pacific Islander, and 6 (3%) as Other. Participant’s ages ranged from 18 to 31 (*M* = 19.43, *SD* = 1.57). A power analysis conducted using G^∗^Power (Faul et al., 2007) assuming a small to medium effect derived from Study 1 (*r* = 0.20) indicated that 191 participants would be needed to achieve 80% power using an alpha level of 0.05 (two-tailed).

### Analyses

To test our competing hypotheses about the relationship between technology use and nonverbal decoding skill, we first examined bivariate correlations between our study variables. Next, we ran a series of linear regressions on the whole sample in Study 1 and Study 2 controlling for participant gender to examine the independent contribution of active and passive technology use on each of our nonverbal decoding skill measures (accuracy scores on the WIPS test, accuracy scores on the DANVA, and self-reported emotional sensitivity).

To combine results from Study 1 and Study 2, a mini meta-analysis (Goh et al., 2016) was performed for each technology use variable and each nonverbal decoding variable. We used fixed effects in which the mean effect size (i.e., mean correlation) was weighted by sample size. All correlations were Fisher’s *z* transformed for analyses and converted back to Pearson correlations for presentation.

## Results

### Study 1

Means, standard deviations, and bivariate correlations are presented in Table 1. Contrary to what would be predicted by either theoretical framework, screen time use was unrelated to every measure of nonverbal decoding skill we employed. However, when examining the ways in which participants self-reported spending their time online, a more complex pattern emerged. Specifically, more active technology use was related to higher self-reported nonverbal decoding skill (*r* = 0.20, *p* < 0.001) but lower accuracy score on the WIPS (*r* = −0.17, *p* < 0.001). That is, participants who identified as more active users (i.e., posting frequently) believed that they were *better* judges of others’ nonverbal communication, but performed significantly *worse* on an objective test of nonverbal decoding skill (i.e., the WIPS test). On the other hand, participants who reported being more passive users (i.e., reading through posts and looking at other people’s photographs) were significantly *more accurate* in decoding nonverbal behavior, as measured by the WIPS (*r* = 0.14, *p* = 0.005), although they did not self-report any differences in their nonverbal decoding skills from less passive users as highlighted by the correlation between passive user endorsement and self-reported skill on the ES subscale of the SSI (*r* = 0.04, *p* = 0.484). Neither self-reported passive nor active technology use was significantly related to an individual’s ability to decode facial expressions of emotions, measured via the DANVA-2AF (*p*’s > 0.07).

**TABLE 1:** Study 1 and study 2 means, standard deviations, and bivariate correlations between technology use, nonverbal decoding skill, and gender.

**Variable**	***M* (*SD*)**		**2**		**3**		**4**		**5**		**6**		**7**	
	**Study 1**	**Study 2**	**Study 1**	**Study 2**	**Study 1**	**Study 2**	**Study 1**	**Study 2**	**Study 1**	**Study 2**	**Study 1**	**Study 2**	**Study 1**	**Study 2**
DANVA 2-AF	0.75 (0.11)	0.74 (0.13)	0.30***	0.42***	0.05	0.11	0.09	0.11	0.03	0.01	0.09	0.10	0.16***	0.30***
WIPS test	0.75 (0.11)	0.74 (0.13)			0.03	0.21**	0.00	−0.03	−0.17***	-0.16*	0.14**	0.27***	0.15**	0.22**
Emotional sensitivity subscale	85.56 (16.93)	87.93 (17.49)					0.02	0.17*	0.20***	0.25***	0.04	−0.03	0.15**	0.35***
Screen time (minutes)	297.88 (136.24)	363.40 (176.50)							0.11^†^	0.24**	0.01	−0.04	0.08	0.12
Active use	4.28 (2.81)	4.00 (2.55)									−0.15**	−0.36***	0.26***	0.23**
Passive use	8.25 (3.05)	8.50 (3.07)											0.02	-0.08
Gender	Male *N* = 210 Female *N* = 196	Male *N* = 92 Female *N* = 98												

*^†^p < 0.10, ^∗^p < 0.05, ^∗∗^p < 0.01, ^∗∗∗^p < 0.001. Study 1 N’s range from 263 to 410 and Study 2 N’s range from 129 to 190 depending on screen time use measure as not all participants had iPhones or screen time turned on. DANVA 2-AF is the Diagnostic Analysis of Nonverbal Accuracy-Adult Faces; Nowicki and Duke (1994). WIPS test is the Workplace Interpersonal Perception Skill test; Dael et al., in preparation. Emotional Sensitivity is a self-reported subscale from the Social Skills Inventory (Riggio, 2005). Active and passive use were measured on 0–10 Likert scale from “does not describe me well” to “describes me very well.” Gender coded as (0 = male, 1 = female).*

### Gender, Technology Use, and Nonverbal Decoding Skill

Because active and passive technology use were not mutually exclusive (i.e., an individual could report being high on active and passive use), and because gender is related to both technology use (Jackson et al., 2008) as well as nonverbal decoding skill (Hall and Gunnery, 2013), we wished to determine the independent effects of active and passive technology use on nonverbal decoding skill while controlling for gender. Therefore, we first entered active use, passive use, and gender into a regression predicting accuracy scores on the WIPS. Active use remained a significant negative predictor (β_*std*_ = −0.21, *p* < 0.001; Table 2), suggesting that those who are more active users were worse at decoding nonverbal behavior. Passive use also remained a significant positive predictor (β_*std*_ = 0.11, *p* = 0.02), where those who reported spending their time looking at others’ posts and pictures were more accurate in decoding nonverbal behavior. Further, these two effects were significant even after controlling for gender, which also significantly predicted higher scores on the WIPS test (β_*std*_ = 0.21, *p* < 0.001; female coded as 1, male coded as 0). Approximately 8% of the variance in WIPS test scores was accounted for when active use, passive use, and gender were entered as predictors.

**TABLE 2:** Regression results from study 1 and study 2 examining the independent contribution of technology use variables on nonverbal decoding skill.

**Study 1**			
**Predictors**	**Objective**		**Self-report**
		
	**DV: WIPS test β_*std*_*t* (*p-*value)**	**DV: DANVA-2AF β_*std*_*t* (*p*-value)**	**DV: Emotional sensitivity β_*std*_*t* (*p*-value)**
Active use	**−0.21 −**4.17 (*p* < 0.001)	**−**0.01 **−**0.16 (*p* = 0.871)	**0.18** 3.51 (*p* < 0.001)
Passive use	**0.11** 2.31 (*p* = 0.021)	0.09 1.77 (*p* = 0.077)	0.06 1.12 (*p* = 0.264)
Gender	**0.21** 4.14 (*p* < 0.001)	**0.17** 3.24 (*p* = 0.001)	0.10 1.95 (*p* = 0.052)
*R*^2^	*R*^2^ = 0.084; *F*(3, 401) = 12.17, *p* < 0.001	*R*^2^ = 0.035; *F*(3, 401) = 4.81, *p* = 0.003	*R*^2^ = 0.051; *F*(3, 401) = 7.17, *p* < 0.001

**Study 2**			

**Predictors**	**DV: WIPS test β_*std*_*t* (*p*-value)**	**DV: DANVA-2AF β_*std*_*t* (*p*-value)**	**DV: Emotional sensitivity β_*std*_*t* (*p-*value)**

Active use	**−**0.13 **−**1.73 (*p* = 0.085)	**−**0.02 **−**0.23 (*p* = 0.815)	**0.21** 2.76 (*p* = 0.006)
Passive use	**0.25** 3.42 (p = 0.001)	0.12 1.59 (*p* = 0.114)	0.06 0.88 (*p* = 0.382)
Gender	**0.27** 3.93 (*p* < 0.001)	**0.32** 4.44 (*p* < 0.001)	**0.31** 4.42 (*p* < 0.001)
*R*^2^	*R*^2^ = 0.15; *F*(3, 188) = 10.87, *p* < 0.001	*R*^2^ = 0.11; *F*(3, 188) = 7.46, *p* < 0.001	*R*^2^ = 0.16; *F*(3, 188) = 11.41, *p* < 0.001

β*_std_ is standardized Beta. Bolded values reflect significance at the p < 0.05 level.*

We next entered active use, passive use, and gender into a regression predicting accuracy scores on the DANVA-2AF. None of these variables, apart from gender (β_*std*_ = 0.17, *p* = 0.001), significantly predicted scores on the DANVA-2AF (Table 2). Approximately 4% of the variance in DANVA-2AF scores was accounted for by these predictor variables.

When active use, passive use, and gender were entered into a regression predicting self-reported nonverbal decoding skill, active use remained a significant positive predictor (β_*std*_ = 0.18, *p* < 0.001), such that those who were more active users self-reported that they were better at decoding nonverbal information from others (Table 2). While more passive use was unrelated to self-reported nonverbal decoding skill, gender remained a marginally significant positive predictor (β_*std*_ = 0.10, *p* = 0.052) indicating that females reported being more skilled nonverbal decoders than males. Approximately 5% of the variance in self-reported nonverbal decoding skill was accounted for when active use, passive use, and gender were entered as predictors.

### Study 2

While results from Study 1 were neither supportive of an enhancing or suppressing effect of global technology usage on nonverbal decoding skill, we did find that the ways individuals used technology mattered (i.e., actively versus passively). Because this active/passive relationship was not hypothesized *a priori*, we examined these effects in a separate sample of participants. Therefore, akin to Study 1, we first examined the bivariate correlations between our measures of technology use and nonverbal decoding skill. We once again found that screen time use was unrelated to objective measures of nonverbal decoding skill—i.e., the DANVA and WIPS (*p’s* > 0.20). However, in Study 2 objective screen time use was significantly and positively related to self-reported nonverbal decoding skill (*r* = 0.17, *p* = 0.050) (Table 1).

Replicating Study 1’s findings, active technology use was also related to higher self-reported nonverbal decoding skill (*r* = 0.25, *p* = 0.001), but lower objective nonverbal decoding skill as measured by the WIPS (*r* = −0.16, *p* = 0.028). Individuals who identified as more passive users were once again significantly more accurate in decoding nonverbal behavior, as measured by the WIPS (*r* = 0.27, *p* < 0.001), although they did not self-report any differences in their nonverbal decoding skills from less passive users (*r* = −0.03, *p* = 0.653). Neither self-reported passive nor active technology use was significantly related to an individual’s ability to decode facial expressions of emotions, measured via the DANVA-2AF (*p’s* > 0.167).

We deconstructed these effects by entering active use, passive use, and gender into three separate linear regressions predicting the WIPS, DANVA-2AF, and self-reported nonverbal decoding skill. We regressed our three predictor variables on scores from the WIPS. Replicating regression results from Study 1, active technology use was a marginally significant negative predictor of nonverbal decoding skill (β_*std*_ = −0.13, *p* = 0.085), passive use remained a significant positive predictor of nonverbal decoding skill (β_*std*_ = 0.25, *p* = 0.001), and gender was a significant predictor, with females scoring higher on the WIPS test compared to males (β_*std*_ = 0.27, *p* < 0.001). This model accounted for 15% of the variance in WIPS scores.

Next, we regressed active use, passive use, and gender on scores from the DANVA-2AF. Once again, gender was the only significant positive predictor (β_*std*_ = 0.32, *p* < 0.001), with females scoring significantly higher than males. Approximately 11% of the variance in DANVA-2AF scores was accounted for by these three predictors.

When active use, passive use, and gender were entered into a regression predicting self-reported nonverbal decoding skill, active use was a significant positive predictor, similar to Study 1, (β_*std*_ = 0.21, *p* = 0.006), such that those who were more active technology users self-reported having more skill in decoding nonverbal information. Reporting more passive technology use was unrelated to self-reported nonverbal decoding skill. Gender remained a significant positive predictor (β_*std*_ = 0.31, *p* < 0.001) indicating that females self-reported more nonverbal decoding skill than males. Approximately 16% of the variance in self-reported nonverbal decoding skill was accounted for when active use, passive use, and gender were entered as predictors.

### Mini Meta-Analysis

Finally, we conducted a mini meta-analysis (Goh et al., 2016) in order to provide a consistent account regarding the relationship between technology use and objective and self-reported measures of nonverbal decoding skill across these two studies. After combining these effects across both studies, we found that individuals who self-reported more active technology use self-reported higher nonverbal decoding skill (M*r* = 0.22, *p* < 0.001), but scored lower on one objective index of nonverbal decoding skill (i.e., the WIPS test: M*r* = −0.17, *p* < 0.001). Moreover, individuals who self-reported more passive use scored significantly higher on both objective indices of nonverbal decoding (i.e., the WIPS test: M*r* = 0.18, *p* < 0.001 and the DANVA2-AF: M*r* = 0.09, *p* = 0.023), but did not self-report higher levels of nonverbal decoding skill (M*r* = 0.02, *p* = 0.667; Table 3).

**TABLE 3:** Mini meta-analysis results from study 1 and study 2 examining combined correlations between measures of technology use and nonverbal decoding skill.

	**Objective**				**Self-report**	

	**WIPS test**		**DANVA-2AF**		**Emotional sensitivity**	
	**M*r* (SE)**	**Combined *Z* [95% CI]**	**M*r* (SE)**	**Combined *Z* [95% CI]**	**M*r* (SE)**	**Combined *Z* [95% CI]**
Screen time (minutes)	−0.01 (0.05)	−0.19 [-0.11, 0.09]	0.10^†^ (0.05)	1.90 [0.00, 0.19]	0.02 (0.05)	0.34 [−0.08, 0.12]
Active use	−0.17*** (0.04)	−4.09 [−0.24, −0.09]	0.02 (0.04)	0.57 [−0.06, 0.10]	0.22*** (0.04)	5.33 [0.14, 0.30]
Passive use	0.18*** (0.04)	4.47 [0.10, 0.26]	0.09* (0.04)	2.27 [0.01, 0.17]	0.02 (0.04)	0.43 [−0.06, 0.10]

*Mr = weighted mean correlation (converted from rz to r). SE is standard error of mean r. ^†^p < 0.10, ^∗^p < 0.05, ^∗∗∗^p < 0.001, all two-tailed.*

## Discussion

While many have theorized about the potential positive or negative effects that technology may have on communication skills, no studies to date have empirically examined the relationship between technology use and nonverbal decoding skill. In order to begin to understand the ways in which technology use and nonverbal decoding skill are related, we measured multiple facets of each construct to more thoroughly examine their empirical relationships with one another.

While overall screen time was unrelated to any measure of nonverbal decoding skill, interesting and consistent patterns emerged when looking at the way individuals spent their time using technology. Specifically, individuals who reported actively posting and engaging with technology-mediated communication self-reported that they were more accurate at decoding the nonverbal behaviors of others. However, these more active users were more likely to score lower on objective measures of nonverbal decoding skill. Conversely, individuals who reported spending their time online passively viewing others’ posts and photos scored higher on objective nonverbal decoding skill but did not self-report that their skills were any better.

These findings lend support to the role of practice and feedback as an effective way to increase nonverbal decoding skill (Blanch-Hartigan et al., 2012). Passive users of communication technology likely receive practice in decoding nonverbal cues simply by being exposed to other users’ content (e.g., pictures, posts, videos) and thus a greater frequency of nonverbal cues. Indeed, the average screen time reported across both studies was about 5 h a day, meaning that passive users may spend up to 5 h each day practicing decoding nonverbal cues. In contrast to “other-focused” passive users, active users likely lose out on a plethora of communication cues as they report spending their time online engaging in “self-focused” activities. That is, although active users likely receive a great deal of practice *encoding* their own thoughts, feelings, attitudes, etc., they do not receive this same practice when it comes to *decoding* the thoughts, feelings, attitudes, etc. of others.

Therefore, these results support both the hypothesis that technology use enhances nonverbal decoding skill, and the hypothesis that technology use worsens nonverbal decoding skill. The key lies in *how* one spends their time using technological platforms. Those who use technology to practice making judgments of others may benefit from time online and learn skills to enhance their face-to-face interactions. However, greater technology use may have the opposite effect for those who choose to spend their time online creating and posting their own content, instead of interacting with the content of others. In these cases, technology may have adverse effects on an individual’s nonverbal decoding skill in face-to-face interactions.

The current research is not without limitations. First, we are limited by our homogenous sample of college participants in one US state. More research is needed to see if the relationship between active and passive technology use and nonverbal decoding skill will generalize more broadly. In addition, while the WIPS test has many advantages to other tests of nonverbal decoding ability (e.g., good reliability and validity, real-world workplace context, dynamic stimuli, many domains of nonverbal sensitivity), it is not yet a published, validated test of decoding ability. Additionally, although self-reporting active and passive technology use provides valid information regarding the way participant’s view their online activity, or the way they are motivated to be, future studies should confirm these self-reports with objective measures in order to assess the accuracy of individual’s self-perceptions. We also examined one aspect of technology use on smartphone devices and the questions focused on self-reported social media use. The role of other technology-mediated communication platforms, such as teleconferencing or interactive video gaming, deserve future study. In our regression models, only 4–16% of the variance in decoding skills was explained by our predictors; therefore, there are many other factors that impact decoding skill ability which should be explored in future work. While the WIPS test is not validated yet (i.e., in prep), it is more ecologically valid than many other available standardized tests of decoding ability because it includes many workplace scenarios and dynamic video rather than focusing on one domain (e.g., emotion recognition like the DANVA-2AF) or using just static photographs where participants often show a ceiling effect on accuracy. In addition, and explained extensively below, we cannot make causal claims about the direction of the relationships given that our data was cross-sectional.

### Suggestions to Further Theories of Technology Use and Nonverbal Decoding Skill

Although our data suggest that the way in which an individual communicates with technology may impact nonverbal decoding skills globally (i.e., as measured by the WIPS test), we only observed a marginally significant effect to suggest that technology use was related to an individual’s ability to decode facial expressions of emotion measured via the DANVA-2AF. While it may be that technology truly does not impact this facet of nonverbal decoding skill, it is also possible that we did not measure technology use at a detailed enough level to reveal any meaningful relationships. Although participants reported technology use generally, different social media and technology communication platforms are vastly different in their bandwidth and each emphasize distinct cue channels. For example, while some platforms emphasize visual cues (e.g., Instagram, Snapchat) others may underscore more verbal cues (e.g., Facebook, Twitter). Collapsing technology use across all platforms may dilute interesting relationships between particular social media apps, cue channels, and nonverbal decoding skill. For instance, it may be that individuals who passively use applications which highlight posting pictures or videos receive more practice in decoding facial expressions, and therefore may score higher on emotion decoding tests such as the DANVA-2AF. Therefore, we urge future researchers to be thoughtful in selecting the most relevant nonverbal decoding skill measure for their particular study Stosic and Bernieri (in prep) taking into account domain (e.g., emotion recognition or general workplace decoding skills) as decoding ability does not appear to be a single skill (Schlegel et al., 2017a), and to further explore the ways in which specific technology-mediated platforms, opposed to global technology use, impact vital communication skills.

In addition to delineating more precise constructs, the areas of technology and nonverbal communication research would benefit from an increase in experimental designs. While we have interpreted our data as technology use potentially influencing nonverbal decoding skills, it is highly plausible that the causal relationship is reversed. Individuals who are more accurate perceivers of others’ nonverbal behavior may be more likely to use technology in a passive way because they are more practiced, more comfortable, or more engaged with others. Those who are less accurate perceivers of others’ nonverbal behavior may use technology more actively because they are more self-focused or find perceiving others to be more challenging or less rewarding. The correlational nature of the current studies does not allow us to untangle the direction of these effects. Therefore, we urge future work to consider experimental designs to examine the causal relationship between technology use and communication ability, particularly nonverbal decoding skill.

While experimental designs on this topic are rare, we are aware of one study that employed a quasi-experimental design to manipulate technology use. Age-matched cohorts of preteens attended a summer camp in a staggered order such that one group went earlier than the other group (Uhls et al., 2014). While at camp, electronics including television, computers, and mobile phones were not allowed. The first group to attend camp was the experimental group (*N* = 51) and the group that stayed at school while the first group was at camp was considered the control group (*N* = 54). After just 5 days of interacting face-to-face without the use of any technology, preteens’ recognition of nonverbal emotion cues from photographs and videos (using the DANVA-2 Child and Adult Faces and the Child and Adolescent Social Perception Measure) was significantly greater compared to the control group. From this, we can gather that the short-term effects of increased opportunities for face-to-face interaction, combined with time away from screen-based media and digital communication, improved preteens’ understanding of and ability to decode nonverbal emotion cues.

Completely removing technology can be difficult in a real-world context; however, there are a variety of methods we propose to untangle the relationship between technology use and nonverbal decoding skill. There are applications and settings on most smartphones that display an alert when the user has reached a screen time maximum for the day. Researchers could consider a dose-response experiment in which they randomly assign different allowed hours of screen time to users each day for a series of days. One could then understand if different doses of screen time lead to higher or lower levels of nonverbal decoding skill.

In another potential research design, researchers could randomly assign the way technology is used by participants. Researchers could assign individuals as “passive users” who are not allowed to post but must read through others’ posts and/or photographs. Some questions to consider are whether or not this would facilitate practice, contribute to learning, and improve nonverbal decoding skill. Another quasi-experimental design could follow emerging adolescents with or without phones and assess differences in their nonverbal decoding skills, accounting for covariates and confounders such as gender, socioeconomic status, parents’ educational levels, and baseline communication skills.

In addition to experimentally manipulating technology use, research could examine and potentially rule out the reverse causality claim that nonverbal decoding skill is driving technology use. To do this, researchers could train participants on nonverbal decoding skill using validated trainings, such as the Geneva Emotion Recognition Test training (GERT; Schlegel et al., 2017b), and then assess whether technology use changes over time or if training nonverbal decoding skill makes technology-mediated communication smoother or more rewarding.

## Conclusion

As the use of technology-mediated communication continues to expand, it is crucial for psychological research to address the positive and negative consequences of technology use on communication skills, in particular nonverbal communication. The current research suggests that it may not be the technology use itself, but rather how actively or passively users engage with technology, that facilitates or hinders nonverbal decoding skill. We ultimately found support for all hypotheses (i.e., Liberated Relationship Perspective, Internet Enhanced Self Disclosure Hypothesis, Reduction Hypothesis, and Cues Filtered Out Theory) but the ways in which the hypotheses were supported depended on how users interacted with technology. Our results showed that those who use technology in a more passive way (reading and look at others’ posts) had higher nonverbal decoding accuracy. That is, more passive users may benefit from time online and learn skills to enhance their face-to-face communication (supporting the Liberated Relationship Perspective and Internet Enhanced Self Disclosure Hypothesis). For those who reported more active use (creating and posting their own content), they had lower nonverbal decoding accuracy. For these more active users, technology may have adverse effects on their ability to read and respond to others in face-to-face communication (supporting the Reduction Hypothesis and Cues Filtered Out Theory).

We believe these results to be encouraging, as some of the fears regarding the negative impact of technology on an individual’s communication skills may not come to fruition if technology is used in a more passive, observational manner rather than an active, self-focused manner. Beyond these results, we also provide researchers with suggestions to further the field of technology use and communication skills. Due to the growing diversity in technology-mediated communication platforms, we urge researchers to account for the different functions theses platforms afford users. In addition, and perhaps most importantly, we urge researchers to explore experimental designs to determine causal pathways in the complex relationship between technology and communication skills. Researchers are beginning to understand how the technological revolution is changing the ways in which humans navigate social interactions. A deeper appreciation for this complexity can lead to the development of interventions to enhance and not hinder our communication skills with the increasing presence and benefits of technology in our lives.

## Data Availability Statement

The raw data supporting the conclusions of this article will be made available by the authors, without undue reservation.

## Ethics Statement

The studies involving human participants were reviewed and approved by the University of Maine IRB. The patients/participants provided their written informed consent to participate in this study.

## Author Contributions

MR, MS, and JC contributed to conception, design of the study, and wrote the first draft of the manuscript. MR organized the database and performed the statistical analysis. DB-H wrote sections of the manuscript. All authors contributed to manuscript revision, read, and approved the submitted version.

## Conflict of Interest

The authors declare that the research was conducted in the absence of any commercial or financial relationships that could be construed as a potential conflict of interest.
